# A Facultative Electroactive Chromium(VI)-Reducing Bacterium Aerobically Isolated From a Biocathode Microbial Fuel Cell

**DOI:** 10.3389/fmicb.2018.02883

**Published:** 2018-11-26

**Authors:** Xiayuan Wu, Xiaoqian Ren, Gary Owens, Gianluca Brunetti, Jun Zhou, Xiaoyu Yong, Ping Wei, Honghua Jia

**Affiliations:** ^1^Bioenergy Research Institute, College of Biotechnology and Pharmaceutical Engineering, Nanjing Tech University, Nanjing, China; ^2^College of Chemical Engineering, Nanjing Tech University, Nanjing, China; ^3^Environmental Contaminants Group, Future Industries Institute, University of South Australia, Adelaide, SA, Australia

**Keywords:** *Bacillus* sp, microbial fuel cell, Cr(VI) removal, biocathode, electrochemical activity

## Abstract

A facultative electroactive bacterium, designated strain H, was aerobically isolated from the biocathode of a hexavalent chromium (Cr(VI))-reducing microbial fuel cell (MFC). Strain H is Gram-positive and rod shaped (1–3 μm length). 16S rRNA gene analysis suggested that this strain (accession number MH782060) belongs to the genus *Bacillus* and shows maximum similarity to *Bacillus cereus* whose electrochemical activity has never previously been reported. Moreover, this strain showed efficient Cr(VI)-reducing ability in both heterotrophic (aerobic LB broth) and autotrophic (anaerobic MFC cathode) environments. Cr(VI) removal reached 50.6 ± 1.8% after 20 h in LB broth supplemented with Cr(VI) (40 mg/L). The strain H biocathode significantly improved the performance of the Cr(VI)-reducing MFC, achieving a maximum power density of 31.80 ± 1.06 mW/m^2^ and Cr(VI) removal rate of 2.56 ± 0.10 mg/L–h, which were 1.26 and 1.75 times higher than those of the MFC with the sterile control cathode, respectively. This study offers a novel Gram-positive *Bacillus* sp. strain for Cr(VI) removal in MFCs, and shows a facile aerobic isolation method could be used to screen facultative electroactive bacteria.

## Introduction

Hexavalent chromium (Cr(VI)) is considered a priority pollutant due to its carcinogenic and mutagenic effects (Fonseca et al., 2012). Significant amounts of Cr(VI) have been discharged into the environment as a result of inappropriate waste disposal by various industries, posing a tremendous threat to humans (Dong et al., 2013). Recently, an emerging technology for Cr(VI) removal using biocathode microbial fuel cells (MFCs) has been proposed. It can efficiently reduce Cr(VI) to Cr(III) by utilizing electroactive biofilms as biocatalysts in the reductive cathode chamber, and simultaneously harvest electricity during the treatment process (Tandukar et al., 2009; Huang et al., 2010). Thus far, several strategies have been applied to enhance the efficiency of the Cr(VI)-reducing biocathode in MFCs, including electrode modification (Wu et al., 2016, 2017), acclimatization optimization (Wu X. et al., 2015), cathode potential control (Huang et al., 2011a), and operating conditions control (Huang et al., 2011b). However, the electroactive Cr(VI)-reducing bacteria on the cathode that play the key role in this system have not been well studied.

Electrochemically active microorganisms (EAMs) are species that can transport electrons outward from cells to extracellular electron acceptors or/and inward from extracellular electron donors into cells (Logan and Rabaey, 2012; Yong et al., 2014; Jafary et al., 2015; Si et al., 2015). The electrochemical activity of EAMs determines the electron transfer efficiency between electrodes and bacteria (Liao et al., 2015; Sun et al., 2017). Thus, EAMs are of vital importance to the performance of MFCs during both electricity generation and contaminant treatment (Lovley, 2012). Although more than 100 EAMs have been isolated or identified, covering a wide range of genetic groups, investigations into EAMs for biocathodes have been limited (Koch and Harnisch, 2016). Most previous studies focused on the isolation of EAMs from the anode biofilm of MFCs in order to improve electricity production, whereas the isolation of EAMs from the cathode biofilm of MFCs for pollution remediation has seldom been reported (Luo et al., 2013; Nor et al., 2015; You et al., 2018). Chen et al. (2016) isolated and identified a Cr(VI)-reducing bacterium (*Ochrobactrum* sp. YC211) from the vicinity of an electroplate factory, and it was found to produce electricity at the anode of MFCs. But the Cr(VI)-reducing biocathode of MFCs is also a valuable microbial resource for mining novel electroactive Cr(VI)-reducing bacteria for Cr(VI) bioremediation and fundamental studies on the electron transfer mechanism. The isolation of bacteria from the Cr(VI)-reducing biocathode, however, has rarely been investigated.

Moreover, the isolation of EAMs from a bioanode (an anaerobic environment for electricity generation in MFCs) is normally conducted under anaerobic conditions, which is complex and sometimes needs expensive anaerobic workstations (Luo et al., 2013; Nor et al., 2015). Except for the obligatory anaerobic EAMs (e.g., *Geobacter sulfurreducens*), many EAMs are facultative bacteria such as *Shewanella oneidensis*, *Pseudomonas aeruginosa*, and *Rhodoferax ferrireducens* (Luo et al., 2013). For example, Xafenias et al. (2013) firstly cultivated *S*. *oneidensis* MR-1 under aerobic conditions in order to obtain a thick biofilm on the working electrode for subsequent anaerobic Cr(VI)-reducing experiments in MFCs. Therefore, the facile aerobic isolation method could be feasible to screen and culture facultative EAMs from anaerobic bio-electrodes in MFCs, which still requires more research (Fedorovich et al., 2009). Furthermore, some facultative EAMs possess Cr(VI)-reducing ability as well, making them more practical and versatile for environmental bioremediation (Xafenias et al., 2013; Chen et al., 2016). Accordingly, facultative EAMs with Cr(VI)-reducing ability could be obtained from anaerobic Cr(VI)-reducing biocathodes in MFCs by using the simpler and faster aerobic isolation method rather than the traditional anaerobic isolation method.

In the present work, a novel facultative electroactive Cr(VI)-reducing strain was aerobically isolated from the cathode biofilm of a Cr(VI)-reducing MFC. The objective of this study was to characterize and identify this strain by physiological and biochemical methods coupled with 16S rRNA gene analysis. In addition, the Cr(VI)-reducing ability of this strain was evaluated in both heterotrophic (aerobic LB broth) and autotrophic (anaerobic MFC cathode) environments. The electrochemical activity of this strain was also assessed by measuring the electricity generation and cyclic voltammetry (CV) in Cr(VI)-reducing MFCs.

## Materials and Methods

### Bacterial Isolation and Culture

The bacterial strain was isolated from the cathode biofilm of a Cr(VI)-reducing MFC that had operated for 3 months. The original inoculant for the biocathode was anaerobic digester sludge from a municipal wastewater treatment plant (Nanjing, China). The side of the cathode was carefully scraped with an inoculating needle and the material removed was dispersed in 10 mL of sterile water. The bacterial suspension was serially diluted and plated in Luria Bertani (LB) solid medium supplemented with 150 mg/L Cr(VI) (prepared by dissolving K_2_Cr_2_O_7_ in deionized water). Samples were aerobically cultured in a chemostat at 30°C. After 3 days of incubation, the dominant colonies were classified morphologically and then streaked onto another LB-Cr plate to obtain the pure isolate.

### Physiological and Biochemical Characterization

Before determining its physiological and biochemical characteristics, the isolate was precultivated and enriched in liquid LB broth in an incubator under aerobic conditions. The incubator was shaken at 150 rpm and the temperature was set at 30°C. During the culture period, bacterial growth was monitored at definite time intervals by using a UV-Vis spectrophotometer (LAMBDA 950, PerkinElmer, United States) with detection at 600 nm. Physiological and biochemical tests were performed using a Biolog Microstation System (Biolog Inc., United States) and the Gram staining method.

### 16S rRNA Gene Sequencing and Analysis

Total genomic DNA was extracted from the isolated strain using a PowerSoil DNA isolation kit (MoBio Laboratories Inc., United States) according to the manufacturer’s instructions. DNA concentration and purification were determined using a NanoDrop 2000 UV-Vis Spectrophotometer (Thermo Scientific, Wilmington, United States), and DNA quality was checked by 1% agarose gel electrophoresis. The 16S rRNA gene was amplified by PCR using a pair of universal primers 27F (5′-AGA GTT TGA TCC TGG CTC AG-3′) and 1492R (5′-GGT TAC CTT GTT ACG ACT T-3′). PCR solutions contained a mixture of 25 μL PCR Master Mix (Promega, Wisconsin, United States), 1 μL of each primer (10 μM), 1 μL of DNA template, and nuclease-free water to make up the total volume of 50 μL. PCR amplification was carried out in a thermocycler PCR system (GeneAmp 9700, ABI, United States) with an initial denaturation of DNA for 4 min at 94°C, followed by 30 cycles of 30 s at 94°C, 1 min at 55°C, and 2 min at 72°C, and then a final extension for 10 min at 72°C. The 16S rRNA gene sequences were queried against the GenBank and Ribosomal Database Project databases using the BLAST algorithms. Phylogenetic relationships were analyzed by the evolutionary distance matrix calculated using the neighbor-joining method. A phylogenetic tree was constructed with the program MEGA5.

### Cr(VI) Removal Tests in a Heterotrophic Environment

The Cr(VI) removal tests of the isolated strain in a heterotrophic environment were conducted in 100-mL conical flasks containing 70 mL of 40 mg/L Cr(VI)-spiked liquid LB medium for 40 h. The Cr(VI) removal tests were run as an aerobic culture at 30°C and 150 rpm with a certain inoculation concentration (OD_600_ = 1) of the isolated strain. Meanwhile, heat-killed cells of the isolated culture and sterile liquid LB-Cr broth under the same conditions served as the control groups. Samples were withdrawn at regular intervals (20 h) and filtered through 0.45-μm membrane syringe filters prior to Cr(VI) concentration determination. All of the experiments were conducted in triplicate to ensure reproducibility.

### MFC Construction and Operation

**FIGURE 1:**
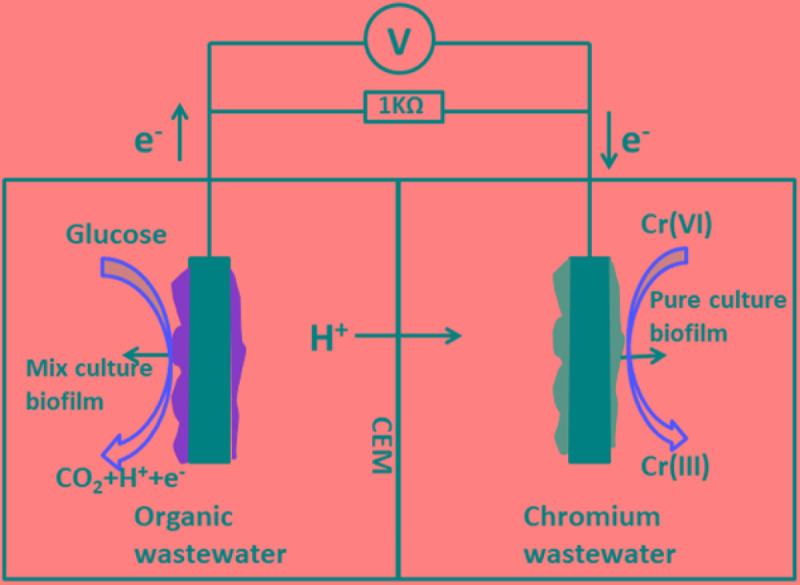
Schematic diagram of the two-chamber microbial fuel cell employed in this study.

The dual chamber MFC was constructed from two plexiglass cubic chambers (liquid volume of 70 mL each) and both chambers were kept gastight. The chambers were separated by a cation exchange membrane (38.5 cm^2^; Nafion117, Dupont Co., United States). A sheet of graphite felt (5.0 cm × 5.0 cm × 0.5 cm) was used as the anode electrode, and also as the cathode electrode. The anode chamber was inoculated with anaerobic digester sludge from a municipal wastewater treatment plant (Nanjing, China). The anolyte consisted of the following: 0.31 g/L NH_4_Cl, 2.452 g/L NaH_2_PO_4_ H_2_O, 4.576 g/L Na_2_HPO_4_, 0.13 g/L KCl, and 1 g/L C_6_H_12_O_6_H_2_O at pH 7.0. During the bioanode acclimatization period, a mixture containing 40 mM ferricyanide and 50 mM phosphate buffer solution (PBS) was used as the catholyte (pH = 7.0). The MFCs were operated in a batch-fed mode at a constant temperature (30°C) connected to an external resistance of 1000 Ω. The schematic diagram of the two-chamber MFC employed in this study is shown in Figure 1.

### Cr(VI) Removal Tests in an Autotrophic Environment (MFC Cathode)

The Cr(VI) removal tests of the isolated strain in an autotrophic environment were performed in the cathode chambers of the MFCs. Sterile graphite felts (5.0 cm × 5.0 cm × 0.5 cm) were placed in 100-mL conical flasks containing 70 mL of liquid LB broth that was inoculated with the isolated strain (OD_600_ = 1). After 20 h of aerobic cultivation at 30°C and 150 rpm, the graphite felts with attached bacteria were taken out from the flasks and gently rinsed with sterile deionized water to minimize carryover of residual organic substances (Wu X. et al., 2015). Subsequently, the graphite felts were transferred to the cathode chambers to anaerobically function as the Cr(VI)-reducing biocathodes in the MFCs. Meanwhile, sterile graphite felts that functioned as abiotic cathodes to reduce Cr(VI) in the MFCs served as the control groups. The pre-acclimatized mature bioanodes with stable potentials were used in the Cr(VI)-reducing MFCs. For the Cr(VI) removal tests, a medium (0.78 g/L KCl, 2.772 g/L NaH_2_PO_4_2H_2_O, 11.53 g/L Na_2_HPO_4_ 12H_2_O, 0.28 g/L NH_4_Cl, 0.2 g/L NaHCO_3_ at pH 7.0) containing an initial Cr(VI) concentration of 27 mg/L was used as the catholyte. The anolyte and other operating conditions were the same as mentioned above. In addition, open circuit controls that were run in the open circuit mode (disconnected electrodes) for Cr(VI) removal were also prepared for the corresponding experimental MFCs.

### Analytical Techniques and Calculations

The voltages generated by the MFCs during the experiments were recorded at 10-min intervals by a data acquisition system (Keithley Instruments 2700, United States). The power density as a function of the current density was obtained from the polarization curve, which was measured at the time point of maximum voltage generation in each MFC; the current and power densities were normalized by the total surface area of the anode. The current (*I*) was calculated using Ohm’s law: *U* = *IR*, where *U* is the voltage and *R* is the external resistance; the power (*P*) was calculated according to *P* = *IU*, while the internal resistance of the MFC was calculated using the polarization slope method (Wu et al., 2016).

Cyclic voltammetry was performed using a potentiostat (CHI660D, Shanghai Chenhua Instruments Co., Ltd., China) in a three-electrode system. The working electrode (cathode) was connected to the counter electrode (anode) at a scan rate of 20 mV/s over the range −600 mV to +600 mV (vs. Ag/AgCl, 195 mV vs. standard hydrogen electrode, SHE).

Biofilm morphology and precipitates on the biocathode were examined using a scanning electron microscopy with coupled energy dispersive spectroscopy (SEM-EDS, Hitachi S-4800, Japan). The bacteria attached to the electrode were stabilized based on previously described procedures (Wu X.-Y. et al., 2015b).

A colorimetric 1,5-diphenylcarbazide method was used for soluble Cr(VI) analysis, and total chromium was analyzed by reoxidizing any reduced form of chromium with potassium permanganate after acid digestion with concentrated HNO_3_ and H_2_SO_4_ (State Environmental Protection Administration, 2002). Samples for soluble Cr(VI) and total chromium analyses were filtered through 0.45-μm membrane syringe filters.

## Results and Discussion

### Isolation, Characterization and Identification of the Cr(VI)-Reducing Bacterium

**FIGURE 2:**
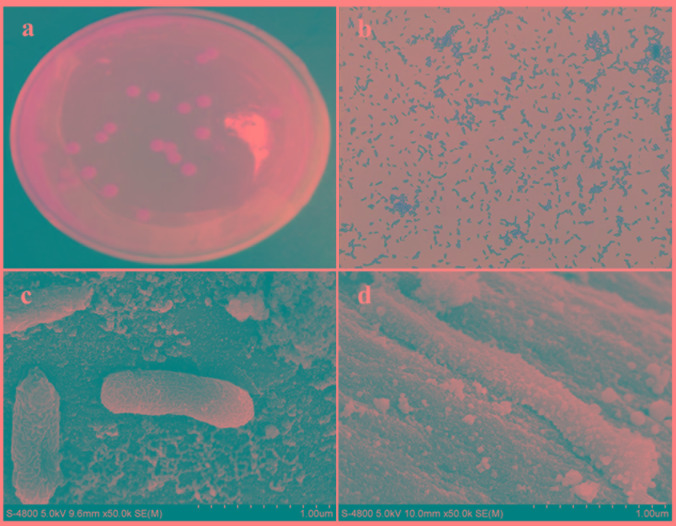
**(a)** Colony image and **(b)** gram staining picture of strain H; scanning electron microscopy images of strain H attached on the graphite felt cathode before Cr(VI) removal **(c)** and after Cr(VI) removal **(d)**.

After 3 days of incubation, only 1 isolate, designated strain H, was obtained from the Cr(VI)-reducing biocathode based on the colony morphology on the plates (Figure 2a). The strain H colony on the LB-Cr solid medium was ivory in color, round and approximately 2–5 mm in diameter (Table 1). The morphology of the isolated strain H was further studied using SEM, and the cells were found to be rod shaped (1–3 μm length) and Gram-positive (Figures 2b,c). The complete details of the biochemical and physiological characteristics of the strain are summarized in Table 1. BIOLOG plates indicated that strain H is able to utilize formic acid, acetic acid, propionic acid, citric acid, L-lactic acid, acetacetic acid, propanetriol, D-mannitol, D-sorbitol, D-arabitol, myo-inositol, glycerol, sodium butyrate, and sodium bromate. In addition, gelatin was hydrolysed and NaCl was tolerated up to a concentration of 8% (w/v).

**Table 1:** Morphological, physiological and biochemical characteristics of strain H (+, positive; −, negative; w, weak).

Test	Result	Test	Result	Test	Result
***Morphological tests***					
Gram’s reaction	+	Colony color	Ivory	Colony surface	Smooth
Cell shape	Rods	Colony shape	Roundness	Colony edge	Regular
Cell length (μm)	1–3	Colony size (mm)	2–5	Colony opacity	Opaque
***Physiological tests***					
pH5	+	1%NaCl	+	8%NaCl	+
pH6	+	4%NaCl	+	Gelatin hydrolysis	+
***Biochemical tests***					
α-D-Glucose	−	Formic acid	+	L-Alanine	w
α-D-Lactose	−	Acetic acid	+	L-Arginine	+
D-Maltose	−	Propionic acid	+	L-Aspartic acid	+
D-Trehalose	−	Citric acid	+	D-Aspartic acid	−
D-Cellobiose	−	L-Lactic acid	+	L-Glutamic acid	+
Sucrose	−	Acetacetic acid	+	L-Histidine	w
Raffinose	−	Propanetriol	+	L-Serine	−
D-Melibiose	−	D-Mannitol	+	D-Serine	w
Dextrin	−	D-Sorbitol	+	Tween 40	−
D-Fructose	−	D-Arabitol	+	Vancomycin	−
L-Fructose	−	Myo-lnositol	+	Lincomycin	−
D-Galactose	−	Glycerol	+	Rifamycin	−
D-Mannose	−	Sodium butyrate	+	Troleandomycin	−
L-Rhamnose	−	Sodium bromate	+	Minocycline	−

**FIGURE 3:**
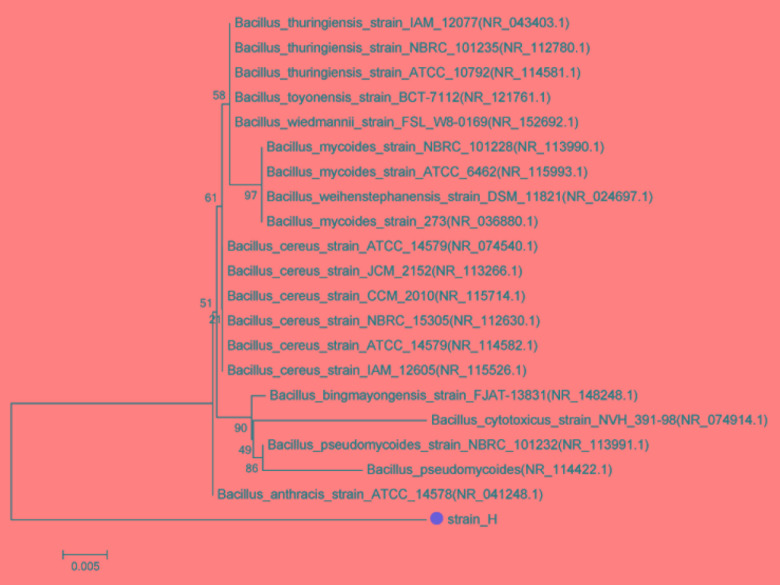
Phylogenetic tree of strain H and closely related species based on 16S rRNA gene sequences. The tree was constructed using the neighbor-joining method.

A 1232-bp target fragment was amplified by PCR using strain H genome DNA as the template and the 16S rRNA gene universal primers 27F/1492R. The sequence found in this study has been deposited in the GenBank database under accession number MH782060. BLAST was applied to find regions of similarity among biological sequences. The neighbor-joining method was used to construct the phylogenetic tree. The results suggested that isolated strain H is a member of the genus *Bacillus* (Figure 3). It was observed that the isolated culture showed maximum similarity (95% sequence similarity) to *Bacillus cereus* strain ATCC14579 (GenBank accession number NR074540.1). *Bacillus* sp. is reported to be actively involved in Cr(VI) reduction in the environment, and many studies have confirmed the Cr(VI)-reducing ability of *B*. *cereus* (Barrera-Diaz et al., 2012; Dong et al., 2013; Murugavelh and Mohanty, 2013). On the other hand, although *B. subtilis* was found to exhibit electrochemical activity for facilitating electron transfer at both the anode and cathode of MFCs (Nimje et al., 2009, 2012; Huang et al., 2011c), the electrochemical activity of *B*. *cereus*, to our knowledge, has never previously been reported in MFCs. Moreover, few studies have focused on the electrochemical activity of Gram-positive bacteria even though they are spread widely across various environments (You et al., 2018). Consequently, whether this isolated Gram-positive *Bacillus* sp. possesses both Cr(VI)-reducing ability and electrochemical activity deserves further study.

### Cr(VI) Removal Using the Isolated Culture in a Heterotrophic Environment

**FIGURE 4:**
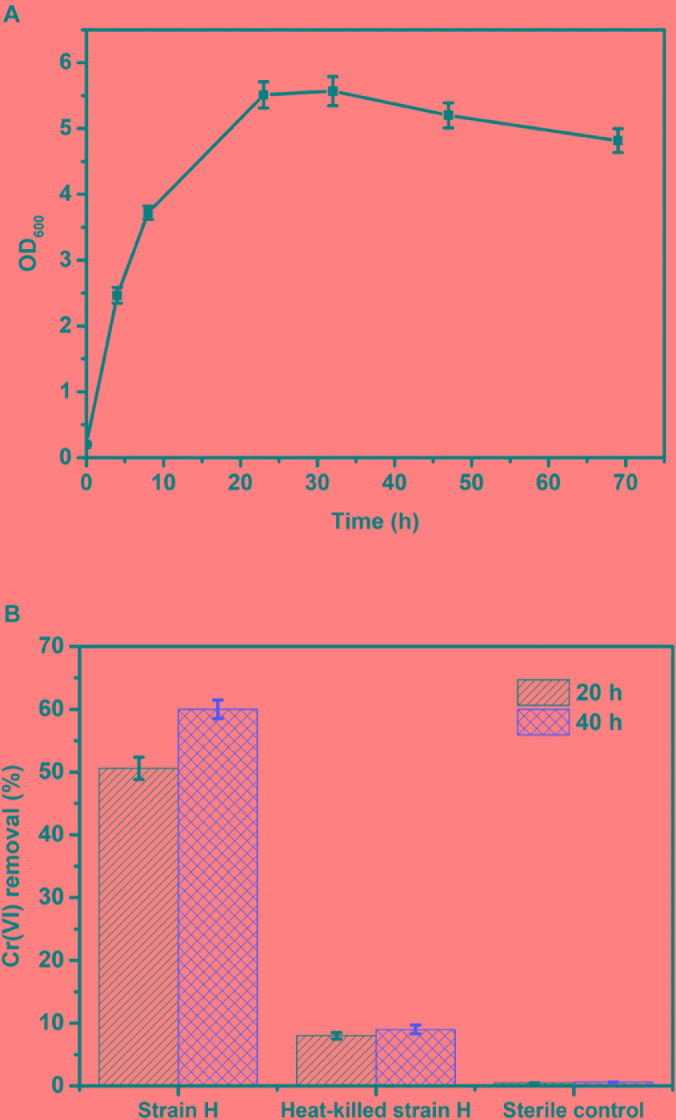
**(A)** Growth curve of strain H in LB liquid broth at pH 7.0 and 30°C; **(B)** Cr(VI) removal of strain H in LB liquid broth at an initial Cr(VI) concentration of 40 mg/L.

The Cr(VI)-reducing ability of isolated strain H in a heterotrophic environment was investigated under aerobic conditions. Before the Cr(VI) removal tests, the growth of strain H in LB liquid broth was monitored at pH 7.0 and 30°C. As shown in Figure 4A, the cell density (OD_600_) of the isolated culture increased rapidly without any lag phase from 0.20 ± 0.02 to 5.51 ± 0.20, implying that the highly active cells directly entered into the logarithmic phase of growth. The logarithmic phase lasted approximately 24 h. Subsequently, after a stationary phase of 10 h, the growth rate of the cells gradually decreased.

Based on the growth curve of strain H, the highly active cells in the logarithmic phase (culture within 24 h) were inoculated for the Cr(VI) removal tests in this study. Figure 4B shows Cr(VI) removal by the isolated strain H in LB liquid broth at an initial Cr(VI) concentration of 40 mg/L. Cr(VI) removal by strain H reached 50.6 ± 1.8% after 20 h, while Cr(VI) removal by the heat-killed cells of strain H was only 8 ± 0.5% and the Cr(VI) concentration was almost unchanged in the sterile control. These results revealed that Cr(VI) bioreduction by strain H rather than Cr(VI) bioadsorption played the key role in Cr(VI) removal. After 40 h, Cr(VI) removal by strain H had slightly increased to 60 ± 1.5%. Since the medium provided sufficient organic electron donors for Cr(VI) reduction, the slight increase in Cr(VI) removal by strain H during the last 20 h might be ascribed to a lower activity of the bacterial cells after the logarithmic phase. This is consistent with the growth curve of strain H, indicating that faster Cr(VI) bioreduction was catalyzed by the highly active cells in the logarithmic phase (within 24 h). A similar phenomenon was reported in an earlier study using an isolated *B. cereus* strain for Cr(VI) reduction (Murugavelh and Mohanty, 2013). Bioadsorption by the heat-killed cells also tended to be saturated at 20 h as there was only a 1% increase in Cr(VI) removal up to the end of the experiments (40 h).

### Cr(VI) Removal Using the Isolated Culture in MFCs

#### Electricity Generation in MFCs

**FIGURE 5:**
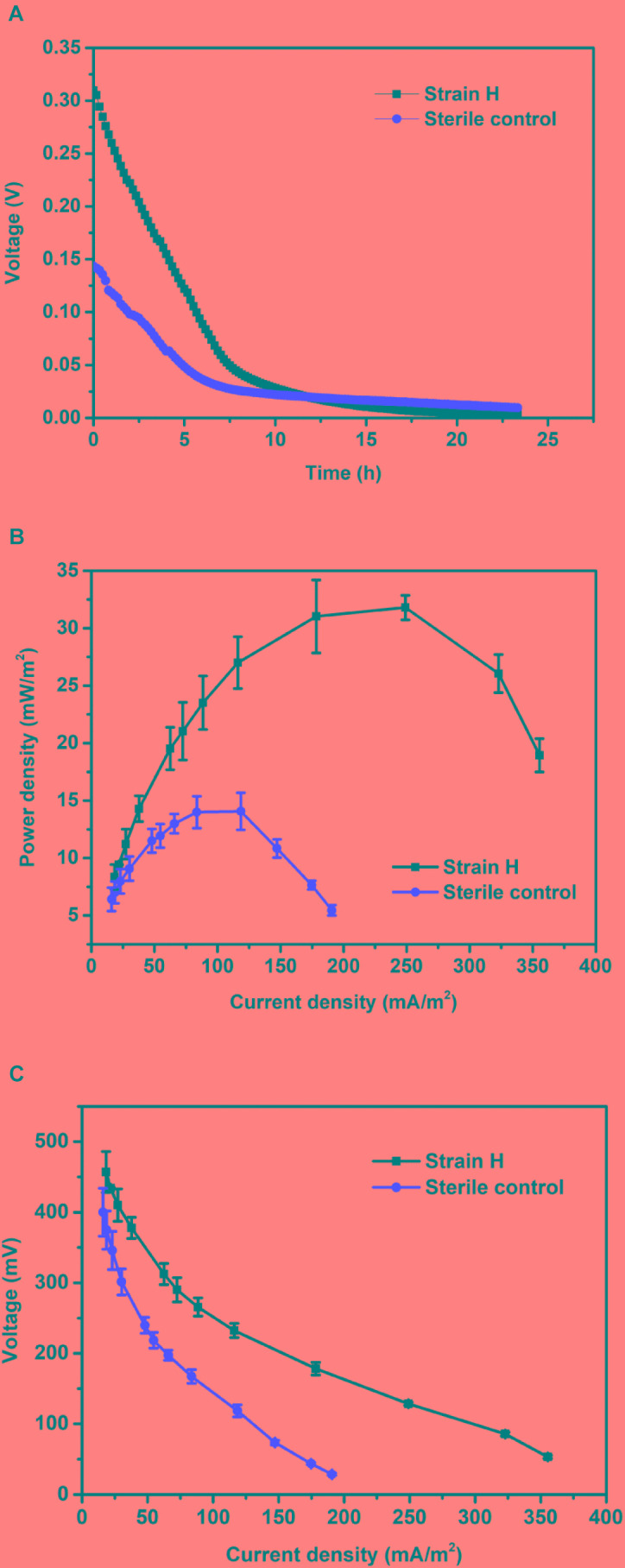
**(A)** Voltage outputs, **(B)** power densities, and **(C)** polarization curves of microbial fuel cells with the strain H biocathode and sterile control cathode.

The Cr(VI)-reducing ability of isolated strain H in an autotrophic environment (MFC cathode) was studied under anaerobic conditions. Typical voltage generation profiles of the Cr(VI)-reducing MFCs are presented in Figure 5A. For Cr(VI)-reducing MFCs, the voltage output normally decreased continuously until the end of the operation, which was closely related to the Cr(VI) concentration in the catholyte (Tandukar et al., 2009; Xafenias et al., 2013; Wu et al., 2016). The maximum voltage of the MFC with the strain H biocathode reached 309.72 mV, which was 1.16 times higher than that of the MFC with the sterile control cathode (143.51 mV). As shown in Figure 5B, the MFC with the strain H biocathode also generated a higher maximum power density of 31.80 ± 1.06 mW/m^2^, which was 1.26 times higher than that of the MFC with the sterile control cathode (14.07 ± 1.61 mW/m^2^). According to the polarization curves (Figure 5C), a lower internal resistance was correspondingly found in the MFC with the strain H biocathode (290.62 ± 26.52 Ω) compared to that of the MFC with the sterile control cathode (393.18 ± 35.71 Ω). These results demonstrated that the presence of strain H on the cathode improved the electricity generation of the Cr(VI)-reducing MFC. This might be attributed to the faster Cr(VI) reduction reaction catalyzed by strain H at the MFC cathode (Tandukar et al., 2009; Huang et al., 2010).

#### Cr(VI) Removal in MFCs

**FIGURE 6:**
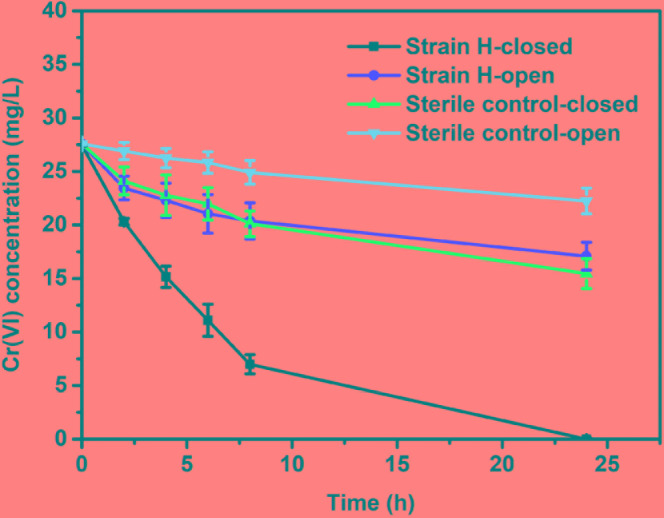
Time course of the dissolved Cr(VI) concentration in microbial fuel cells with the strain H biocathode and sterile control cathode under open and closed circuit conditions.

In order to clarify the Cr(VI) removal mechanisms, the changes in the Cr(VI) concentrations in the MFCs with different cathodes were investigated under both open and closed circuit conditions. As can be seen from Figure 6, the Cr(VI) removal rates of the two cathodes under open circuit conditions were distinctly lower than those observed under closed circuit conditions; in particular, a larger gap in Cr(VI) removal between the two sets of conditions was found for the MFC with the strain H biocathode. Under open circuit conditions, the Cr(VI) removal rate of the strain H biocathode achieved 0.90 ± 0.20 mg/L h after 8 h, which was 1.73 times higher than that of the sterile control cathode (0.33 ± 0.13 mg/L h). Cr(VI) removal at the sterile control cathode was mainly due to physical adsorption on the graphite felt. On the other hand, for the strain H biocathode, on top of the physical adsorption at the electrode, Cr(VI) removal was also caused by bioadsorption and a minimal contribution from the decay of the endogenous biomass upon Cr(VI) reduction (Tandukar et al., 2009; Wu et al., 2016, 2017). Once the circuits were connected, the Cr(VI) concentrations decreased faster in the two MFCs, and especially in the MFC with the strain H biocathode, indicating that the bioelectrochemical process stimulated Cr(VI) removal in the autotrophic cathode environment. Cr(VI) was completely removed in the MFC with the strain H biocathode at the end of operation (24 h). The Cr(VI) removal rate of the MFC with the strain H biocathode reached 2.56 ± 0.10 mg/L h at 8 h, which was 1.75 times higher than that of the MFC with the sterile control cathode (0.93 ± 0.14 mg/L h). The Cr(VI) concentration in the MFC cathodes decreased continuously with time, which was consistent with the electricity generation pattern in Figure 5A. This relationship between Cr(VI) concentration and electricity generation was also observed for Cu(II)-reducing MFCs (Heijne et al., 2010). The Cr(VI) removal rate of the MFC with the strain H biocathode was enhanced by 1.85 times after the circuit was connected, implying that strain H possesses electrochemical activity that catalyzes the Cr(VI) reduction by utilizing the electrons from the cathode in the autotrophic environment. To the best of our knowledge, only pure cultures of *Shewanella* strains have been applied as biocathodes for Cr(VI) removal in MFCs (Hsu et al., 2012; Xafenias et al., 2013). The maximum power density and Cr(VI) removal rate only achieved 2.11 mW/m^2^ and 2.25 mg/L h, respectively, by using *S*. *oneidensis* MR-1 pure culture as the biocathode with the presence of lactate for Cr(VI) removal in MFCs (Xafenias et al., 2013). Therefore, strain H isolated in this study shows a great potential in electrochemical reduction of Cr(VI).

#### Electrochemical and Morphological Analyses of the Biocathode

**FIGURE 7:**
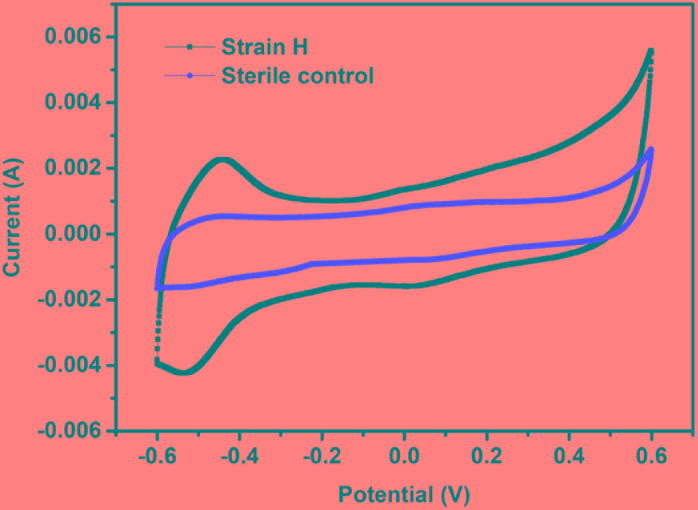
Cyclic voltammograms of the strain H biocathode and sterile control cathode in microbial fuel cells (vs. Ag/AgCl, scan rate of 20 mV/s between –600 mV and +600 mV).

Previous reports have indicated that Cr(VI) reduction at a biocathode of a MFC is largely due to the bioelectrochemical reaction mediated by the cathodic bacteria (Tandukar et al., 2009; Huang et al., 2010; Wu X.-Y. et al., 2015b; Wu et al., 2016, 2017). CV has been proposed as an effective method of investigating microbial activity in MFCs because the magnitude of redox peaks reveals the electrochemical activity of a biofilm (Luo et al., 2013; You et al., 2018). CV was therefore employed to confirm the electrochemical activity of the strain H biocathode at the initial time of MFC operation (Figure 7). A distinct redox couple was observed from the strain H biocathode, whereas no redox peak was detected from the sterile control cathode. For the strain H biocathode, an oxidation peak in the forward scan of the voltammogram appeared at −436 mV (vs. Ag/AgCl) with a current of 2.27 mA, while a reduction peak was found at −543 mV (vs. Ag/AgCl) with a current of 4.24 mA during the reverse scan. These observations demonstrated that strain H on the cathode possesses electrochemical activity that catalyzes Cr(VI) reduction. Another two isolated facultative and Gram-positive bacteria from the genus *Bacillus*, *B. subtilis* and *B. megaterium*, were reported to have electrochemical activity that was mainly due to excreted redox mediators (such as riboflavin), which were responsible for the redox peaks appearing in CV curves (Nimje et al., 2009; You et al., 2018). Similarly, the electrochemical activity of strain H could probably be ascribed to excreted redox mediators as well, but this requires further investigation. On the other hand, *S*. *oneidensis* MR-1, as a pure culture for Cr(VI) reduction at a MFC cathode, also exhibited redox peaks during CV analysis (Xafenias et al., 2013). Thus, this work offers a novel Gram-positive strain for future fundamental studies of the electron transfer mechanism and heavy metal bioremediation in addition to the existing strains such as *Shewanella* and *Geobacter* species (Gregory and Lovley, 2005; Xafenias et al., 2013).

**FIGURE 8:**
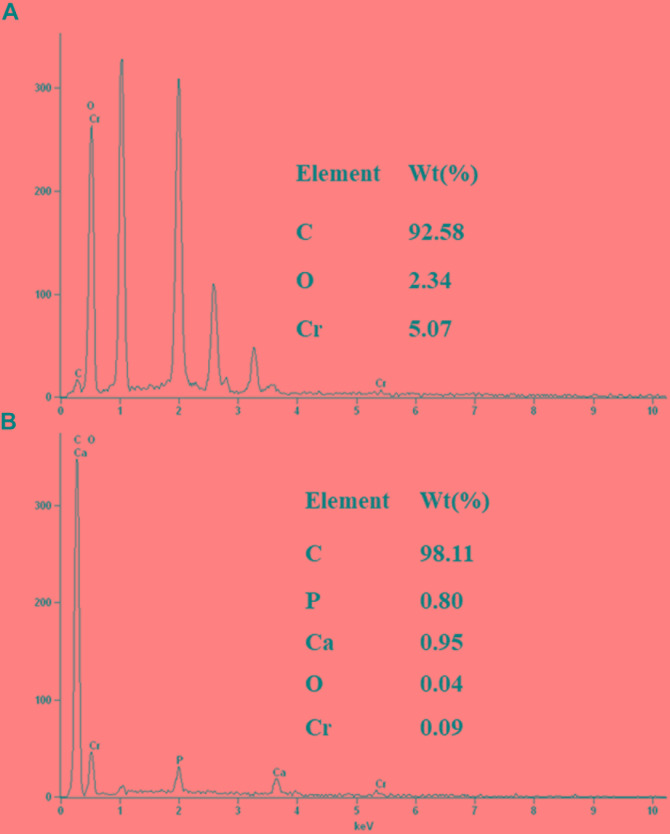
Energy dispersive spectroscopy spectra of the precipitates on the surface of the graphite felt cathode **(A)** and the surface of strain H bacterial cells **(B)** after Cr(VI) removal.

The morphology of the strain H biocathode was observed using SEM before and after Cr(VI) removal (Figures 2c,d). Compared with the strain H biocathode before Cr(VI) removal, some noticeable precipitates were found on the surface of the graphite felt cathode and the bacterial cells after the strain H biocathode was used for Cr(VI) removal. According to the EDS analysis (Figure 8) and previous research results, these precipitates were Cr(VI) reduzates mainly composed of Cr(OH)_3_ (Xafenias et al., 2013; Wu et al., 2016). The SEM-EDS results revealed that strain H successfully reduced Cr(VI) and produced Cr(III) oxyhydroxide deposits extracellularly in both anaerobic and autotrophic MFC cathode environments. The surprising Cr(VI)-reducing ability of strain H in both environments suggests that there is a tremendous, practical potential for the treatment and remediation of Cr(VI) pollution using biological methods. Furthermore, the results of this study imply that a facile aerobic isolation method could be applied to screen facultative electroactive and efficient Cr(VI)-reducing bacteria, providing a new insight into Cr(VI) bioelectro-remediation. Meanwhile, as compared to other electroactive Gram-positive *Bacillus* sp. strains in previous studies (Table 2), strain H produced the highest power density in the MFC cathode for Cr(VI) reduction. Most electroactive *Bacillus* sp. strains were applied in the MFC anode or/and for nitrate reduction, and *B*. *subtilis* is the most frequently reported. It means other *Bacillus* sp. strains rather than *B*. *subtilis* still remain unexploited. This study offers a novel electroactive *Bacillus* sp. strain to explore.

**Table 2:** List of electroactive Gram-positive *Bacillus* sp. strains in studies.

Microbe	Experimental system and location	Power density (mW/m^2^)	Pollutant removal ability	Reference
*Bacillus megaterium* LLD-1	MFC (anode)	30.36	NA	(You et al., 2018)
*Bacillus subtilis* BBK006	MFC (anode)	1.05 × 10^4^	NA	(Nimje et al., 2009)
*Bacillus subtilis* BBK006	MFC (cathode)	19	Nitrate reduction	(Nimje et al., 2012)
*Bacillus subtilis* moh3	MDC (anode)	3.1	Waste engine oil degradation	(Sabina et al., 2014)
*Bacillus* sp.	MFC (anode)	1.14	Nitrate reduction	(Zhang et al., 2012)
*Bacillus* sp. H	MFC (cathode)	31.80	Cr(VI) reduction	This study

*MFC, microbial fuel cell; MDC, microbial desalination cell; NA, not applicable.*

## Conclusion

A facultative electroactive bacterium, *Bacillus* sp. H, was isolated from the biocathode of a Cr(VI)-reducing MFC. This strain showed efficient Cr(VI)-reducing ability in both heterotrophic (LB broth) and autotrophic (MFC cathode) environments. Cr(VI) removal reached 50.6 ± 1.8% after 20 h in LB broth supplemented with Cr(VI) (40 mg/L). The strain H biocathode significantly improved the performance of the Cr(VI)-reducing MFC, obtaining a maximum power density of 31.80 ± 1.06 mW/m^2^ and Cr(VI) removal rate of 2.56 ± 0.10 mg/L h, which were 1.26 and 1.75 times higher than those of the MFC with the sterile control cathode, respectively.

## Author Contributions

XW and HJ designed the experiments. XW and XR performed the experiments. JZ and XY helped XW in the analysis of the data. XW drafted the manuscript. PW contributed to the revision of the manuscript. GO and GB contributed to the English language editing.

## Conflict of Interest Statement

The authors declare that the research was conducted in the absence of any commercial or financial relationships that could be construed as a potential conflict of interest.
